# Prevalence of Human papillomavirus (HPV) and Epstein-Barr virus (EBV) in oral and oropharyngeal squamous cell carcinoma in south-eastern Poland

**DOI:** 10.1186/s13027-015-0031-z

**Published:** 2015-10-12

**Authors:** Dorota Polz-Gruszka, Kamal Morshed, Agnieszka Stec, Małgorzata Polz-Dacewicz

**Affiliations:** Department of Virology, Medical University of Lublin, ul. Chodźki 1, 20-093 Lublin, Poland; Chair and Department of Otolaryngology and Laryngological Oncology, ul. Jaczewskiego 8, 20-054 Lublin, Poland

**Keywords:** Epstein-Barr virus, Human papillomavirus, Oral and oropharyngeal squamous cell carcinoma (OSCC), Co-infection

## Abstract

**Objective:**

The aim of this study was to analyze the prevalence of HPV and EBV in oral and oropharyngeal squamous cell carcinoma in south-eastern Poland. The correlation between viral infection, OSCC, alcohol use, tobacco smoking, demographic data (gender, age, place of residence), anatomic location, pre-treatment staging, evidence of metastases to lymph nodes, and grading was also investigated.

**Methods:**

The examination samples were collected from paraffin tissue blocks, from 154 patients. Viral DNA was amplified by the nested-PCR method.

**Results:**

HPV DNA was detected in 29.2 % of the tested samples (in 27.4 % of oropharyngeal and in 30.4 % of oral cavity). The HPV type 16 was detected in 15.6 % of all samples, and in 53.3 % of HPV-positive group. In HPV-positive samples from oropharyngeal HPV 16 constitute 76.5 %, and in HPV-positive samples from oral cavity HPV 16 constitute 39.3 %. Mixed infection (more than one type of HPV) was observed in 23.5 and 60.7 %, respectively, and in 46.7 % of all HPV-positive samples, and in 12.3 % of the whole study group. EBV DNA was detected in 27.3 % of the cases and HPV-EBV co-infection in 7.8 % of samples.

**Conclusions:**

In major patients from Southeastern region of Poland with oropharyngeal cancer HPV type 16 was detected but in oral cavity cancer other mixed infections were observed (i.e. 51, 52, 59, 66, 68, 71, 74). HPV 16 was detected more often among patients younger than 50 years of age, whereas the mixed HPV in the group aged 50–59. The pathogenesis of oral squamous cell carcinoma may be connected with EBV infection. Future studies on the mechanisms of HPV/EBV co-infection and/or superinfection and their role in oral squamous cell carcinoma are necessary.

## Introduction

Oral squamous cell carcinoma (OSCC) is a serious public health problem in many parts of the world and in Poland too. In Poland oral and oropharyngeal cancer constitutes 3.8 % cancers among men and 1.3 % cancers among women [1]. Oral and oropharyngeal squamous cell carcinoma (OSCC) in tumor histology accounts for more than 90 % of the cases [2]. The etiology of OSCC is considered to be multifactorial and the factors influencing it include environmental factors, life style, infection agents and genetic alterations. The main predisposing factors are tobacco and alcohol abuse [3]. An important role in the etiology of many of them is played by the oncogenic viruses [2]. The cancerogenecity of HPV in humans was conducted by the International Agency for Research on Cancer (IARC) in 2007 and 2012 [4, 5].

The first original observation that implicated HPV as a risk factor in the development of oral cancer was presented by Syrjänen et al. in 1983 [6]. Since then, several studies have focused on HPV detection in oral cancer [7]. A more recent study by Syrjänen and Syrjänen showed a strong association between the presence of HPV DNA, specifically HPV16, and OSCC [8]. This meta-analysis showed that HPV significantly increases the risk for OSCC, as compared with controls. HPV positive oropharyngeal cancer varied according to the geographical region, i.e. in North America – 56 %, in Japan 52 %, in Australia 45 %, in Northern and West Europe 38 % [9].

Epstein-Barr virus, which is also known as human herpesvirus 4 (HHV-4) and which belongs to the *Herpesviridae* family, has a double-stranded DNA genome [10]. EBV is an enveloped virus with icosahedral capsids symmetry and the genome takes on a linear form in mature virions and a circular episomal form during the period of latency in the infected cells. This is one of the most common viruses in humans [11]. EBV was the first human virus to which the oncogenic potential was attributed, and it is classified as group 1 carcinogen by the World Health Organization’s International Agency for Research on Cancer [12]. EBV is correlated with nasopharyngeal and gastric carcinoma, squamous cell carcinoma, Hodgkin’s lymphoma, and Burkitt’s lymphoma [10, 13–15]. Current investigations suggest that EBV is correlated with many diseases localized in the oral cavity such as gingivitis, periodontitis, pulpitis, periapical inflammations and periodontal abscesses [14, 16]. A lot of studies indicate co-infection with HPV and EBV viruses in oral squamous cell carcinoma [17, 18].

The aim of this study was to analyze the prevalence of HPV and EBV and their co-infection in oral and oropharyngeal squamous cell carcinoma in south-eastern Poland.

## Material and methods

### Sample collection

Tissue samples were obtained from 154 patients with primary oral and oropharyngeal SCC treated in the Chair and Department of Otolaryngology and Laryngological Oncology of the Medical University of Lublin. Samples were collected in 2006–2009. The group consisted of 131 males and 23 females, aged between 40 and 87 (average 56.8 years). In the examination group there were 92 patients with oral, and 62 with oropharyngeal SCC. It was a retrospective investigation and the examined materials were obtained from formalin-fixed, paraffin-embedded tissues. The samples were collected during surgery, but TNM was calculated during primary diagnosis (T-tumor, N-nodus, M-metastasis). One hundred percent of the patients were classified as N0, M0. The patients were treated surgically with or without postoperative radiotherapy, depending on the clinical stage of the disease. The patients did not receive radiotherapy or chemotherapy before. TNM classification was done according to the criteria of the Union Against Cancer (UICC) [19]. Histological grading was performed according to the World Health Organization criteria, which divide tumors into three types: well differentiated (G1), moderately differentiated (G2), and poorly differentiated (G3) [20].

This research was approved by the Ethics Committee and it was is in accordance with the GCP regulations (no. KE-0254/181/2012).

### DNA extraction from paraffin sections

We used 5 × 10-μm sections of formalin-fixed, paraffin-embedded tissues from OSCC samples. DNA was extracted using a protocol as described in the DNeasy Tissue Kit Handbook (Qiagen GmBH, Hilden, Germany, cat. No 69506). Purified DNA was quantified by spectrophotometry (Epoch Microplate Spectrophotometr, BioTek Instruments Inc., Vinooski, Vermont USA).

### EBV DNA detection

The nested PCR was carried out for detection of EBV DNA. The product size was 54 bp. The primer sequences are:

Outer

5′ – GTC ATC TAC GGG GAC ACG GA – 3′

5′ – AAG AAG AGA TAT GTG GGG GT – 3′

Inner

5′ – ACC CGG AGC CTG TTT GTG GC– 3′

5′ – GGA GAA GGT CTT CTC GGC CTC – 3′

All PCR reactions were carried out in the final volume of 25 μl using Taq PCR Core Kit (Qiagen, Hilden, Germany). Concentrations of PCR reaction components were prepared as follows: 2 mM MgCl_2_, 0.2 mM dNTPs, 0.2 μM of each forward and reverse primers and 0.5 U of Taq DNA polymerase. During each run the samples were tested together with one negative and one positive control. The negative control consisted of nuclease-free water, positive - EBV-positive cell line, Namalwa, ATCC-CRL-1432. The reaction mixture containing 5 μl of extracted DNA was amplified under the following conditions: 94 °C for 3 min of initial denaturation, then 35 cycles of 94 °C for 30 s, 55 °C for 40 s, 72 °C for 1 min with the final extension at 72 °C for 5 min. The second round amplification was performed with 1 μl of the outer product in the same conditions. The final PCR products were analysed on 3 % agarose gel.

**HPV DNA detection**; HPV detection and genotyping was performed using the INNO-LiPA HPV Genotyping Extraassay (Innogenetics N. V, Gent, Belgium; no cat. 81063) according to the manufacturer’s protocol. The kit is based on the amplification of a 65 bp fragment from the L1 region of the HPV genome. PCR products are subsequently typed with the reverse hybridization assay. The assay covers all currently known high-risk HPV genotypes and probable high-risk HPV genotypes (16, 18, 26, 31, 33, 35, 39, 45, 51, 52, 53, 56, 58, 59, 66, 68, 73, 82) as well as a number of low-risk HPV genotypes (6, 11, 40, 43, 44, 54, 70) and some additional types (69, 71, 74).

### Statistical analysis

Statistical analysis was performed to investigate the relationship between EBV and HPV presence and clinical and demographical characteristics of patients were determined by means of Pearson’s chi-square test and with Fisher’s exact test for small groups. Statistical significance was defined as *p* < 0.05.

## Results

**Fig. 1:**
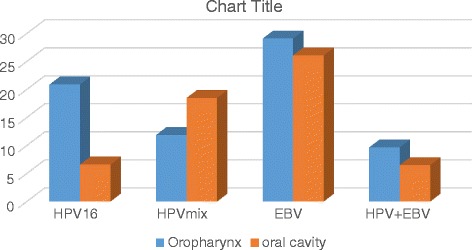
Prevalence of HPV, EBV and HPV/EBV coinfection in oropharyngeal and oral cavity cancer (%)

Males (85.1 %) with, smoking (85.0 %) and alcohol abuse (64.3 %) problems prevailed in the studied group. The majority of the tumors in the examination group were classified as G2 (81.8 %). T4 (58.4 %) and N2 (50.0 %) traits occurred in the majority of patients. No cases of metastasis were observed (M0 100 %). Characteristics of the study group are shown in Table 1. In the Fig. 1 the prevalence of HPV, EBV and HPV/EBV co-infection were presented.

**Table 1:** Epidemiological and clinical characteristics of patients

		Oropharynx		Oral cavity		Total	
		*N* = 62	%	*N* = 92	%	*N* = 154	%
Sex	female	6	9.7	17	18.5	23	14.9
	male	56	90.3	75	81.5	131	85.1
Age	40–49	12	19.3	23	25.0	35	22.8
	50–59	46	74.2	60	65.2	106	68.8
	70 +	4	6.5	9	9.8	13	8.4
Place of residence	urban	37	59.7	55	59.8	92	59.7
	rural	25	40.3	37	40.2	62	40.3
Smoking	yes	52	83.9	79	85.9	131	85.1
	no	3	4.8	5	5.4	8	5.2
	No answer	7	11.3	8	8.7	15	9.7
Alkohol abuse	yes	42	67,7	57	62.0	99	64.3
	No	7	11.3	6	6.5	13	8.4
	No answer	13	21.0	29	31.5	42	27.3
Histology stage G	1	2	3.2	12	13.0	14	9.1
	2	50	80.6	76	82.6	126	81.8
	3	10	16.2	4	4.4	14	9.1
T stage	1	0	0	0	0	0	0
	2	2	3.3	31	33.7	33	21.4
	3	12	19.4	19	20.7	31	20.2
	4	48	77.4	42	45.6	90	58.4
N stage	No	11	17.7	19	20.6	30	19.5
	N1	14	22.6	18	19.6	32	20.8
	N2	35	56.5	42	45.7	77	50.0
	N3	2	3.2	13	14.1	15	9.7
M stage	0	62	100.0	92	100.0	154	100.0
	1	0	0	0	0	0	0

Of all 154 samples, 29.2 % (45/154) were HPV positive, 27.4 % (17/62) in oropharyngeal and 30.4 % (28/92) in oral cavity cancer. HPV type 16 was detected in 20.9 % (13/62) of oropharyngeal and in 12 % (11/92) of oral cancer. The HPV type 16 was detected in 53.3 % of HPV-positive group; in oropharyngeal more often (76.5 %) than in samples from the oral cavity (39.3 %). This difference is statistically significant (*p* < 0.01). Other mixed infections (more than one type of HPV i.e. 51, 52, 59, 66, 68, 71, 74) were detected in 23.5 and 60.7 % of the cases, respectively, and in 46.7 % of all HPV-positive samples.

Of all investigated samples, 27.3 % were positive for EBV, in oropharyngeal 29.1 % and in oral cavity 26.1 %. Co-infection HPV/ EBV was detected in 7.8 % (12/154), in oropharyngeal 9.7 % (6/62) and in the oral cavity 6.5 % (6/92). EBV and HPV 16 co-infection was detected in 6 cases i.e. 50.0 % of co-infected samples. There are no correlation between HPV or EBV infection and age and tobacco smoking (Table 2). Table 3 shows the prevalence of HPV and EBV in different histopathological grades and TN classification. Among HPV-positive patients G1 was diagnosed more often (15.6 %) than among HPV-negative ones (6.4 %), and G3 more often in HPV-negative. There is a statistically significant (*p* < 0.05).

**Table 2:** HPV 16, HPV other and EBV by age and smoking

Age	HPV 16	HPV mix	EBV
	*N* = 24	*N* = 21	*N* = 42
40–49	8 (22.9 %)	6 (17.1 %)	5 (14.3 %)
*N* = 35			
50–59	13 (12.3 %)	15 (14.2 %)	33 (31.4 %)
*N* = 106			
70+	3 (23.1 %)	0	4 (30.8 %)
*N* = 13			
*p*	>0.05	>0.05	>0.05
Smoking	20 (83.3 %)	15 (71.4 %)	32 (76.2 %)
Yes			
*N* = 131			
No	4 (16.7 %)	6 (28.6 %)	10 (23.8 %)
*N* = 23			
*p*	>0.05	>0.05	>0.05

**Table 3:** Prevalence of HPV and EBV in different histopathological grades and TN classification

	HPV+ *n* = 45	HPV- *n* = 109	EBV+ *n* = 42	EBV- *n* = 112	*p* value
G1	7 (15.6)	7 (6.4)	4 (9.5)	10 (8.9)	>0.05
*n* = 14					
G2	37 (82.2)	89 (81.7)	34 (81.0)	92 (82.2)	
*n* = 126					
G3	1 (2.2)	13 (11.9)	4 (9.5)	10 (8.9)	
*n* = 14					
*P*	<0.05*		>0.05		
T1	0	0	0	0	>0.05
*n* = 0					
T2	7 (15.6)	26 (23.8)	6 (14.3)	27 (24.1)	
*n* = 33					
T3	9 (20.0)	22 (20.2)	8 (19.0)	24 (21.4)	
*n* = 31					
T4	29 (64.4)	60 (55.0)	28 (66.7)	61 (54.5)	
*n* = 89					
*p*	>0.05		>0.05		
No	6 (13.3)	24 (22.0)	26 (61.9)	4 (3.6)	>0.05
*n* = 30					
N1	6 (13.3)	26 (23.9)	7 (16.7)	25 (22.3)	
*n* = 32					
N2	25 (55.6)	52 (47.7)	7 (16.7)	70 (62.5)	
*n* = 77					
N3	8 (17.8)	7 (6.4)	2 (4.7)	13 (11.6)	
*n* = 15					
*p*	>0.05		>0.05		

*statistical significant

## Discussion

The prevalence of HPV in squamous cell carcinoma of the oral and oropharynx is diverse and ranges from 8 to 74 % [21–24]. This is the first original observation about frequency of HPV and EBV in oral and oropharyngeal cancer among Polish patients. In our study HPV DNA was detected in 29.2 % of the tested samples (27.4 % - oropharynx; 30.4 % - oral cavity) and is lower than in Northern and Western Europe. Poland is located in East Europe, so prevalence of HPV be able to different than in other European countries. HPV infection is a sexually transmitted infection, so sexual behaviors can play a role.

Studies by Robinson et al. [25] did not reveal any association between the histological grade and HPV status in these tumours. Instead, Zhao et al [26] by multivariate analysis have found a correlation between HPV infection and poor histological grade (OR = 104.0, 95 % CI: 11.2–962.1). His study provides evidence that the presence of HPV predicted a better survival.

In the present study a significant relationship was found between the status of HPV and the poor histopathological grades (chi square, *p* < 0.05; Table 3). In most studies no association was found between HPV presence and TNM clinical stage [26]. Our results are similar.

Confirmation or exclusion of HPV DNA in OSCC influences prognosis and therapy choices. Some researchers state that OSCC patients who are HPV-positive have a more favorable treatment prognosis [20–22], higher survival rates and a lower risk of the disease recurrence in comparison to the HPV-negative patients [27]. Additionally, such patients do not require radio or chemotherapy afterwards [28], and according Khode [29] HPV-positive tumors are more sensitive to chemotherapy.

Although squamous cell carcinoma has been commonly believed to affect people above fifty, the newest data indicate that the age of people afflicted with this disease is constantly decreasing [30]. The study by Golusiński et al. [30] on the occurrence of this disease in people under 45 years of age indicates that these cases constitute up to 0.24–9.0 % of all cases, and that this problem is relatively new. These data show that oral and oropharynx cancers are common among people over 50; however, they also occur at a younger age (80.8 and 19.2 %, respectively) [31]. Among patients younger than 50 HPV 16 was detected more often (22.9 %) than in the group aged 50–59 (12.3 %). In patients 70 years and over was only 3 HPV positive cases (Table 2).

Smokers prevailed among patients infected both with HPV and EBV. It was found out that the largest number of smoking patients were in the group infected with HPV 16 virus (83.3 %; Table 2). Probably, there is a correlation between smoking and HPV 16 infection on the one hand and oropharyngeal squamous cell carcinoma, on the other [32]. Merne et al. [33] suggest a possible synergy between tobacco components and viral oncogenes, especially HPV16 E6/E7 in transformation of oral epithelial cells. Studies by Haukioja et al. [34] found out that smoking is a risk factor for a persistent oral HPV infection. On the other hand, persistent HPV infection in the oral mucosa might increase the risk of developing oral cancer [35].

A number of authors emphasize the role of EBV in the development of OSCC [14, 15, 20].

Zur Hausen drew attention as early as 1976 to the possibility of the involvement of EBV in human cancer [36]. Jaloluli et al. [18] observed the presence of EBV in 55 % of samples from 8 different countries. The frequency of occurrence of EBV virus varies in different populations. Opinions on the role of EBV in OSCC are different. Literature provides a wide range of preponderance for oral, pharynx and larynx viral infections. Nevertheless, the obtained research results suggest that the pathogenesis of oral squamous cell carcinoma is not directly connected with *Herpesviridae* infection in the oral cavity [37]. EBV DNA was detected in our research in 27.3 % of the cases (42/154), most often in the 50–59 years old group (31.4 %; Table 2). The frequency of EBV DNA in oropharyngeal cancer was slightly higher 29 % (18/62) compared with the oral cavity – 26.1 % (24/92) (Fig. 1). This low infection rate may result from the fact that formalin-fixed, paraffin-embedded tissues was examined.. The presence of the EBV is most likely connected with more aggressive types of oral tumors, particularly in groups of immunosuppressed patients [38]. Infection with EBV can be of importance since EBV is widespread in the human population (antibodies to EBV have approximately 95 % of adults). The infection may be asymptomatic and life-long [39]. According to Kis et al. [40], the prevalence of Epstein-Barr virus in OSCC patients is significantly higher than in the healthy group and higher than that in other mucosa pathologies such as oral leukoplakia and oral lichen planus (73.8, 19.1, 29.5 and 46.6 %, respectively).

Some authors find a connection between EBV infection (especially co-infection with papillomaviruses) and squamous cell carcinoma of the tongue and oropharyngeal sites [14, 17, 41]. In our study HPV-EBV co-infection was detected only in 7.8 % of samples (Fig. 1). This low percentage can not support that co-infection plays a role in OSCC. However, it cannot be completely exluded. Jalouli et al. [42] studied tissue samples obtained from patients with oral cancers and they showed that prevalence of HPV and EBV infections is common and may influence the oral cancer development. Co-infection by multiple oncogenic viruses may be an important risk factor in the development of OSCC [17, 43]. Jiang et al. [17] suggest that coinfected cells can have a higher tumorigenic potential than normal cells, and that co-infection of both HPV and EBV may have a more profound effect on invasion than proliferation.

The question remains whether in these cases we have to do with co-infection or superinfection. It is known that chronic virus infection affects the immunological answer of the host. Primary infection with non-oncogenic virus can favour superinfection with oncogenic virus capable of cancer transformation. The oncogenic potential of HPV is related to the expression of E6 and E7, the oncogenic potential of EBV – to the expression of LMP-1 and LMP-2. Toll-like receptors (TLRs) play a critical role in the early innate immune response to the invading pathogens by sensing a microorganism and they are involved in sensing endogenous danger signals [44, 45]. Fathallah et al [45] established that the EBV oncoprotein latent membrane protein 1 (LMP1) is a strong inhibitor of TLR9 transcription, which can favour the aforementioned superinfection. A limitation in our studies is a too small number of cases of EBV/HPV, coinfection, which makes the epidemiological analysis of this group of patients impossible.

In conclusion, the present study shows that in major patients from Southeastern region of Poland with oropharyngeal cancer HPV type 16 was detected but in oral cavity cancer other mixed infections were observed (i.e. 51, 52, 59, 66, 68, 71, 74). HPV 16 was detected more often among patients younger than 50 age of years, whereas the mixed HPV in the group aged 50–59. EBV may play a role in the development of OSCC. Future both epidemiological and studies on the mechanisms of co-infection and/or superinfection and their role in oral squamous cell carcinoma are necessary. The knowledge about these mechanisms may provide targets for therapy and for development of diagnostic methods.
